# High Strength Al–La, Al–Ce, and Al–Ni Eutectic Aluminum Alloys Obtained by High-Pressure Torsion

**DOI:** 10.3390/ma14216404

**Published:** 2021-10-26

**Authors:** Stanislav O. Rogachev, Evgeniya A. Naumova, Eva A. Lukina, Adrian V. Zavodov, Vladimir M. Khatkevich

**Affiliations:** 1 Department of Physical Metallurgy and Physics of Strength, National University of Science and Technology MISIS, 119991 Moscow, Russia; jan73@mail.ru; 2 Department of Composite Materials, MSUT “STANKIN”, 127055 Moscow, Russia; 3 All-Russian Scientific-Research Institute of Aviation Materials (VIAM), 105005 Moscow, Russia; lukinaea@viam.ru (E.A.L.); zavodovad@gmail.com (A.V.Z.); 4 Research and Development Center TMK (TMK R&D), 143026 Moscow, Russia; hatvm87@mail.ru

**Keywords:** eutectic aluminum alloys, high-pressure torsion, microstructure, mechanical properties

## Abstract

A comparative analysis of the effect of high-pressure torsion (HPT) on the microstructure and tensile properties of the Al–10% La, Al–9% Ce, and Al–7% Ni model binary eutectic aluminum alloys is carried out. An HPT of 20-mm diameter specimens in as-cast state was carried out under constrained conditions, at room temperature, pressure *P* = 6 GPa, and number of turns *N* = 5. It is shown that the formation of nano- and submicrocrystalline structures and the refinement of eutectic particles in aluminum alloys simultaneously provide a multiple increase in strength while maintaining a high plasticity margin. This combination of properties has been achieved for the first time for severely deformed binary aluminum eutectics. The relationship between the type of eutectic particles, the structure formation process and the mechanical properties of the aluminum alloys has been established. The thermal stability of severely deformed aluminum alloys at heating up to 200 °C has been studied.

## 1. Introduction

Currently, an urgent task is the creation of high-tech aluminum alloys with increased mechanical and special physical properties, for example, a low temperature coefficient of linear expansion, increased wear resistance and/or increased strength at elevated temperatures [1]. The widely used hypereutectic silumins (alloys of the Al–Si system) have a number of significant disadvantages, namely, embrittlement, the need for modification during melting, and reduced thermal conductivity. Developed in recent years, the multicomponent eutectic alloys based on the systems such as aluminum–calcium (light, corrosion-resistant), aluminum–cerium and aluminum–lanthanum (heat-resistant), aluminum–nickel (high-strength and heat-resistant) are very promising for practical use [2,3,4,5,6]. These alloys are highly technological in casting, since they have narrow crystallization intervals, and they are easily deformed in the annealed state, despite the large fraction (over 10% by volume) of intermetallic phases in the structure. The lack of solubility in the equilibrium state fundamentally distinguishes such alloys from other eutectic aluminum alloys, for example, Al–Cu and Al–Si. At the same time, all base (binary) eutectic compositions (Al–Ca, Al–Ce, Al–La, and Al–Ni) without the addition of aluminum solid solution strengthening elements (Zn, Mg, Cu, Zr, Sc) have an average level of strength properties comparable to the properties of silumins. It is of practical interest to increase the strength properties of base eutectic alloys without additional alloying (which leads to a decrease in technological properties and an increase in the cost of alloys), which expands the scope of their use in modern technology.

It is known that the strength properties of aluminum alloys can be increased by deformation methods [7]. A large number of studies are devoted to increasing the strength of pure aluminum and aluminum alloys due to their structure refinement by the severe plastic deformation (SPD) techniques: high-pressure torsion (HPT), equal channel angular pressing (ECAP), rotary forging, etc. [8,9,10,11,12,13,14,15,16,17,18,19,20,21,22]. Today, the HPT technique allows you to process large-scale specimens (20–30 mm diameter), which makes it possible to carry out tensile testing using miniature tensile specimens [23].

The problem of a simultaneous increase in strength while maintaining high plasticity during SPD is well known. There are only a few studies related to obtaining an ultrafine-grained structure in complexly alloyed aluminum alloys containing cerium and lanthanum [24,25], calcium [26] and nickel [27]. So, in work [25], for a complex eutectic alloy Al–5.4% Ce–3.1% La, a sixfold increase in strength was achieved with a twofold decrease in plasticity as a result of HPT. It should be noted that in complex eutectic alloys, chemical elements can affect mutual solubility and cause other effects [26]. Therefore, the behavior of binary eutectic alloys differs significantly from the behavior of multicomponent eutectic alloys, in which there is a more complex mutual influence of the components.

In this work, the 20-mm diameter specimens of the Al–10% La, Al–9% Ce, and Al–7% Ni as-cast model binary eutectic alloys were processed by the HPT technique, and a comparative study of their microstructure and tensile properties was carried out. For a correct comparative analysis, we used double aluminum alloys, the content of the second component in each alloy corresponds to the eutectic composition in accordance with the phase diagram. Despite the different mass fraction of the second component in the three alloys, the volume fraction of the eutectic in all alloys is close, which is also preferable for comparative analysis.

## 2. Materials and Methods

The following two-component cast eutectic aluminum alloys were taken as materials for the study: Al–10% La, Al–9% Ce, and Al–7% Ni (the chemical composition is given here in wt.%).

The melting was carried out in an electric resistance furnace using a graphite-chased crucible and a high purity aluminum (99.99%). Pure cerium and lanthanum were introduced into the aluminum melt, and nickel was introduced as Al–20% Ni alloy. The casting was carried out in a graphite mould at a temperature of ~780 °C to obtain flat ingots with dimensions of 15 × 30 × 180 mm (the cooling rate during solidification was ~10 K/s).

The HPT-deformation was carried out using specimens with a diameter of 20 mm and an initial thickness of 1.5 mm at room temperature, pressure *P* = 6 GPa, and number of turns *N* = 5. The constrained conditions for deformation process have been used, i.e., the Bridgman anvil installation had an upper anvil with a flat base and a lower rotating anvil with a profiled hole 1-mm deep where the specimen was placed [28]. After HPT, the thickness of the specimens was ~1.1 mm.

The microstructure of aluminum alloys before and after HPT was studied by transmission electron microscopy (TEM) using Tecnai G2 F20 S-TWIN equipment with a Schottky-type thermal-field cathode. The analyzed area corresponded to the mid-radius of the disk-like HPT-specimen. The process was carried out using different modes, namely, light and dark modes, and high-resolution mode (HRTEM). The samples (foils) were prepared through the following stages: (1) electrospark cutting, (2) mechanical thinning on SiC paper, and (3) electrolytic polishing using a TenuPol-5 installation at a temperature of minus 40 °C and a voltage of 17 V. Electrolyte composition: CH_3_OH: HNO_3_ = 15: 85 volume parts. After electrolytic polishing, the samples were cleaned from surface contaminants using a PIPS II ion polishing unit in an argon atmosphere at an accelerating voltage of 0.2–0.5 kV. The transverse size of the structure elements (crystallites, particles) was calculated from TEM images using the ImageExpert software. At least 100 structure elements were measured for each state of the aluminum alloy sample.

The phase composition and structure of the samples were studied by X-ray diffractometry using a monochromatic CuKα radiation and a DRON 3M diffractometer. Before X-ray studies the central part of the HPT-specimen was removed, and the specimen was ground to a depth of 1/3 of the disc thickness, followed by polishing of the surface. Analysis of X-ray diffraction patterns and determination of the volume fraction of phases were carried out by the Rietveld method. The calculation of the dislocation density (ρ) was carried out according to the Equation (1):

ρ = δ/(*b* × *D*), (1)

where: δ is the half-width of the interference line; *b* is Burgers vector (0.286 nm); and *D* is the crystallite size.

The strain uniformity of the specimens under HPT has been evaluated by measuring the Vickers’s microhardness (load 0.5 N, holding time 10 s) on two mutually perpendicular diameters of the specimens with a step of 1 mm (3 measurements for each point with a distance between adjacent points of 0.1 mm). Before measuring the microhardness, the specimens were ground to a depth of 1/3 of the disc thickness, followed by polishing the surface. Microhardness measurements were carried out using a Micromet 5101 tester (Buehler, Leinfelden-Echterdingen, Germany).

The tensile tests were carried out using miniature specimens with 12 mm full length and the gage part length, width and thickness of 5, 1.45, and 1 mm, respectively, using an INSTRON 5966 testing machine (Instron, Norwood, MA, USA). Tensile specimens were cut by the electrospark technique, so that their gage part was located on the mid-radius of the disk-like HPT-specimen.

To study the thermal stability, the aluminum alloy samples after HPT were heated in an electric furnace at temperatures of 150 and 200 °C with holding for 1 h and cooled in air, followed by a tensile test.

Fractographic analysis of specimens after tensile tests was carried out using a JSM-IT500 scanning microscope (JEOL Ltd., Tokyo, Japan) at ×30–3000 magnifications. This microscope was also used to study the structure of the HPT-processed specimens. The area near the specimen mid-radius was analyzed.

## 3. Results

### 3.1. Effect of the HPT-Deformation on Microhardness of the Aluminum Alloys

**Figure 1:**
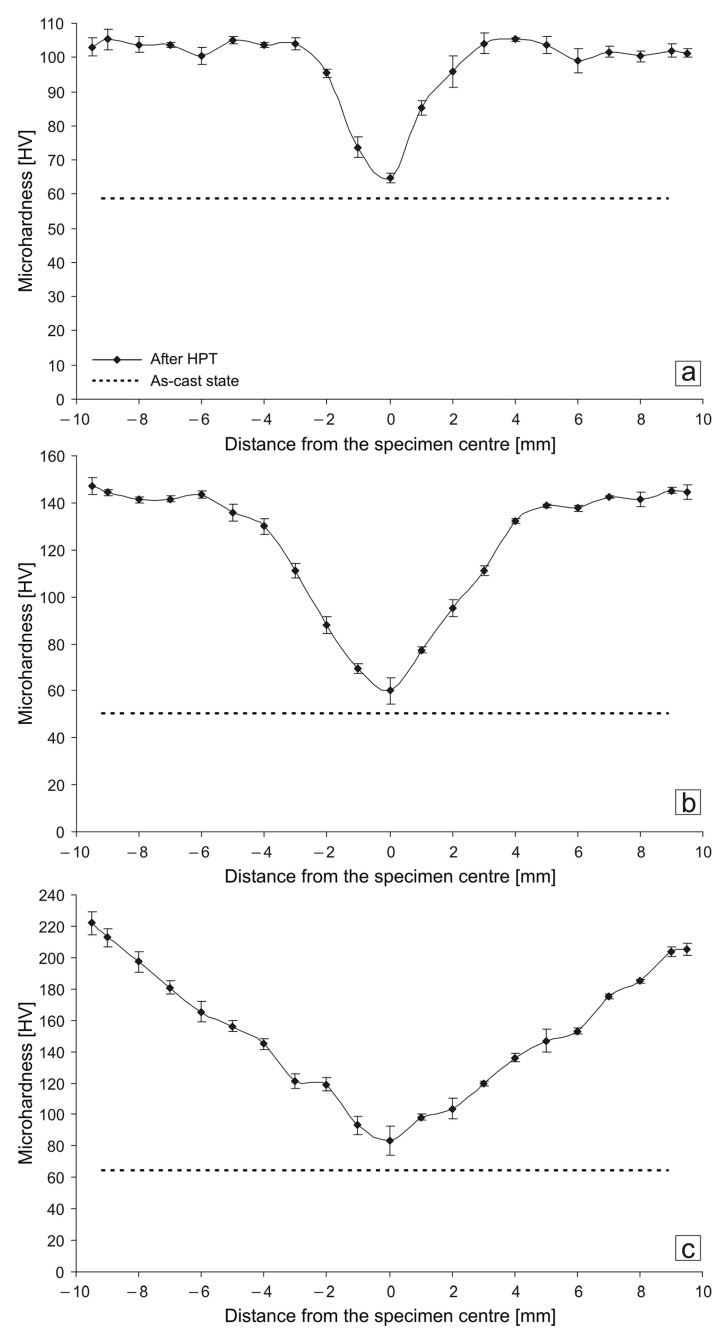
Microhardness distribution along the diameter of the HPT-processed specimens: (**a**) Al–10% La; (**b**) Al–9% Ce; (**c**) Al–7% Ni.

The HPT-deformation of all aluminum alloys leads to a significant increase in the values of microhardness and to the appearance of inhomogeneity of their distribution over the specimen diameter: the minimum values of microhardness were observed in the center of the specimen, and the maximum values were observed at its periphery (Figure 1). The shape of the microhardness value distribution profiles along the specimen diameter differs between all alloys. For example, for the Al–10% La alloy specimen, a ‘dip’ of the microhardness is observed only in the central region 1.5-mm radius, and at a greater distance from the center to the periphery, the microhardness values quickly reach a maximum and remain at a constant level. For the Al–9% Ce alloy specimen, with distance from the center to the periphery, the microhardness values monotonically increase, reach a maximum at a distance of 4 mm from the center, and remain at a constant level. For the Al–7% Ni alloy specimen, a monotonic increase in the microhardness values from the center to the periphery is observed along entire diameter of the specimen (i.e., a gradient of microhardness is observed). Thus, the homogeneity of the microhardness value distribution increases in the following series of alloys: Al–7% Ni, Al–9% Ce, and Al–10% La.

The maximum microhardness values after HPT increase in the following series of alloys: Al–10% La (105–108 HV), Al–9% Ce (145–150 HV), and Al–7% Ni (214–220 HV). The hardening effect after HPT (the ratio of the maximum microhardness value of the alloy after HPT to the average microhardness value of the alloy before HPT) increases in the following series of alloys: Al–10% La (1.8 times), Al–9% Ce (2.8 times), and Al–7% Ni (3.3 times).

### 3.2. Effect of the HPT-Deformation on the Structure of the Aluminum Alloys

**Figure 2:**
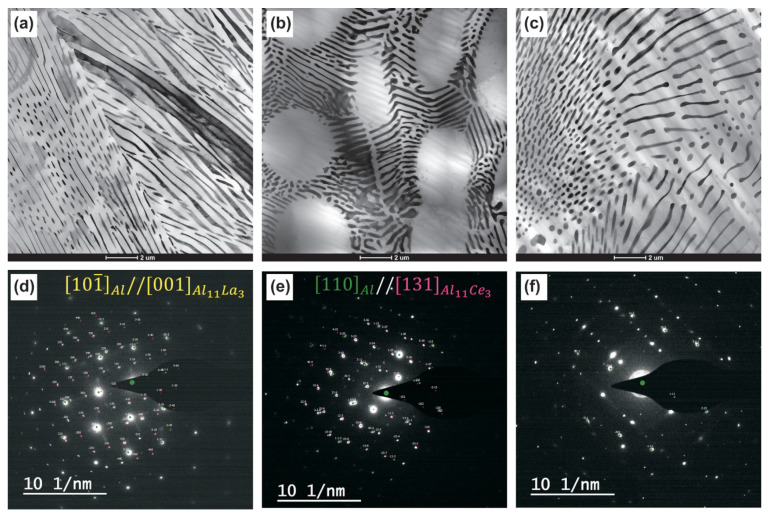
Bright-field TEM images and corresponding SAED patterns of as-cast alloys microstructure: (**a**,**d**) Al–10% La; (**b**,**e**), Al–9% Ce; (**c**,**f**) Al–7% Ni.

According to TEM data, the structure of all aluminum alloys in the initial cast state consisted of an aluminum base (Al) and eutectic, namely, [(Al) + Al_11_La_3_]), [(Al) + Al_11_Ce_3_]), and [(Al) + Al_3_Ni], respectively, in the Al–10% La, Al–9% Ce, and Al–7% Ni alloys (Figure 2). The predominant length ranges of eutectic particles in the Al–10% La, Al–9% Ce, and Al–7% Ni alloys were 3000–10,000 nm, 1000–5000 nm, and 1000–5000 nm, respectively. The predominant thickness ranges of eutectic particles in the Al–10% La, Al–9% Ce, and Al–7% Ni alloys were 40–120 nm, 70–130 nm, and 150–300 nm, respectively. Thus, in the Al–10% La alloy, the eutectic particles are more elongated and thinner in comparison with the Al–9% Ce and Al–7% Ni alloys. In the Al–10% La and Al–7% Ni alloys, the eutectic is evenly distributed in the volume of the metal, and in the Al–9% Ce alloy it is located along the boundaries of aluminum dendrites. The most uniform distribution of eutectic particles was observed in the Al–7% Ni alloy. At the same time, according to SEM data, the Al–7% Ni alloy contained a certain amount of Al_3_Ni large primary crystals.

**Figure 3:**
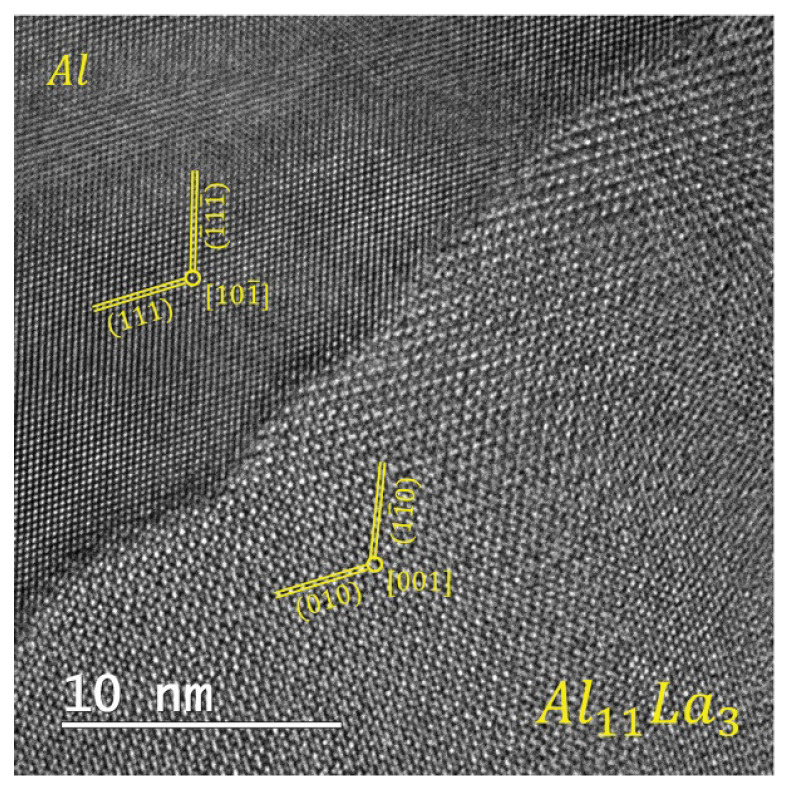
HRTEM image of as-cast Al–10% La alloy microstructure.

According to the TEM analysis data, the as-cast Al–10% La alloy has an ([$10\bar{1}$] Al || [001] Al11La3) orientation relationship between the phases (Figure 3). An explicit orientation relationship is not observed the Al–9% Ce alloy. The orientation relationship between the phases in the Al–7% Ni alloy could not be determined.

**Figure 4:**
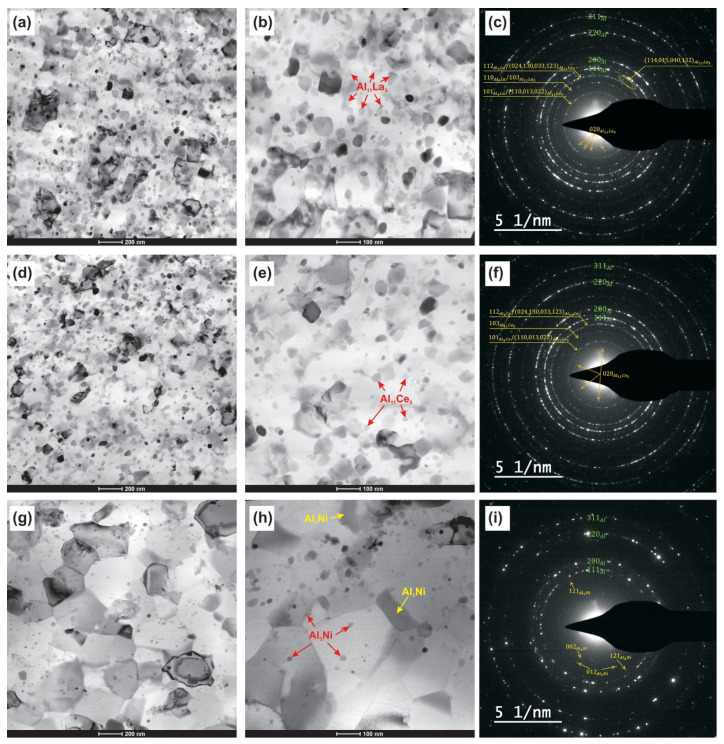
Bright-field TEM images and corresponding SAED patterns of the HPT-processed alloys: (**a**–**c**) Al–10% La; (**d**–**f**) Al–9% Ce; (**g**–**i**) Al–7% Ni.

According to TEM analysis data, the structure of all aluminum samples after the HPT-deformation is represented by an aluminum base and the intermetallic phase particles located both along the grain boundaries and in their volume. HPT led to the formation of nano- and submicrocrystalline structure in the Al–10% La and Al–9% Ce alloys and a submicrocrystalline structure in the Al–7% Ni alloy, as well as to the eutectic particle refinement in all alloys (Figure 4). However, in the HPT-processed Al–7% Ni alloy structure, the individual large fragments of non-crushed eutectic particles up to 600 nm are retained (indicated by a yellow arrow in the Figure 4h). At high magnifications, the boundaries of the crystallites are clearly visible, while the crystallites have a predominantly equiaxed shape with the presence of triple intersections with an angle close to 120°, and the inner region of the crystallites is light without contrast, which indicates a relatively low density of intracrystalline defects in the structure of all alloys after HPT.

A quantitative estimate of the dislocation density based on X-ray analysis data is given in Table 1. The dislocation density is of the same order in all alloys after HPT, while it is the smallest in the Al–7% Ni alloy and the highest in the Al–9% Ce alloy.

The presence of a grain–subgrain structure with a different misorientation of the structure elements in all samples after HPT was judged, firstly, by the presence of point reflections in SAED patterns (from high-angle boundaries), as well as reflections with azimuthal blur (from low-angle boundaries); secondly, by contrast in a series of obtained dark-field images.

The particles in the structure of the samples were identified by the EDS method. The absence of solubility in the aluminum solid solution was judged by EDS, as well as by the absence of changes (within the measurement error) of the interplanar spacing according to the X-ray diffractometry data (Table 1).

**Figure 5:**
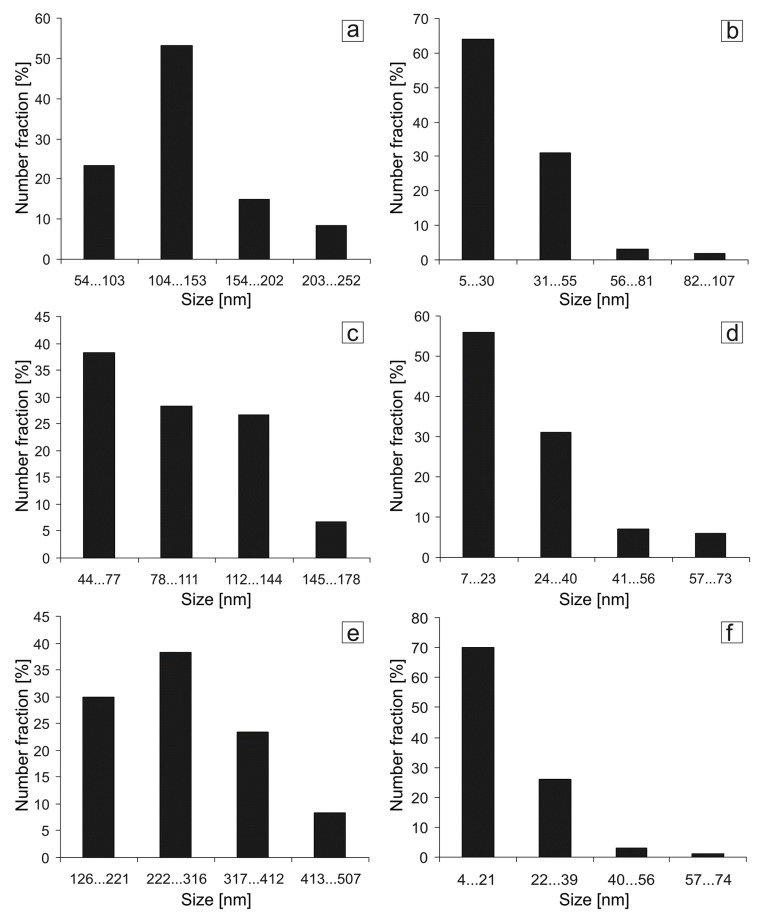
Histograms of size distribution of crystallites (**a**,**c**,**e**) and particles (**b**,**d**,**f**) in the HPT-processed aluminum alloys: (**a**,**b**) Al–10% La; (**c**,**d**) Al–9% Ce; (**e**,**f**) Al–7% Ni.

The crystallite and particle size distribution histograms for the HPT-processed aluminum alloys are shown in Figure 5. The average crystallite sizes in the Al–10% La, Al–9% Ce, and Al–7% Ni alloys were 134 ± 10 nm, 97 ± 8 nm, and 276 ± 24 nm, respectively. At the same time, the predominant crystallite size ranges were 90–150 nm, 60–130 nm, and 170–400 nm, respectively, in the Al–10% La, Al–9% Ce, and Al–7% Ni alloys. Thus, in the Al–10% La and Al–9% Ce alloys, the crystallite size is comparable, and in the Al–7% Ni alloy, the crystallite size is 2–2.8 times larger. The average sizes of eutectic particles after HPT were 28 ± 3 nm, 26 ± 3 nm, and 19 ± 2 nm, respectively, in the Al–10% La, Al–9% Ce, and Al–7% Ni alloys. At the same time, the predominant eutectic particle size ranges were 10–50 nm, 10–40 nm, and 7–25 nm, respectively, in the Al–10% La, Al–9% Ce, and Al–7% Ni alloys. A comparable size of strengthening particles can be observed in alloys, for example, the 7xxx series, treated according to the T7 mode (quenching + slight over-aging) [29]. In a number of studies, it is argued that under the HPT process, the deformed alloy can pass into a phase state corresponding to its equilibrium phase state after prolonged annealing at a certain higher temperature [30,31].

**Figure 6:**
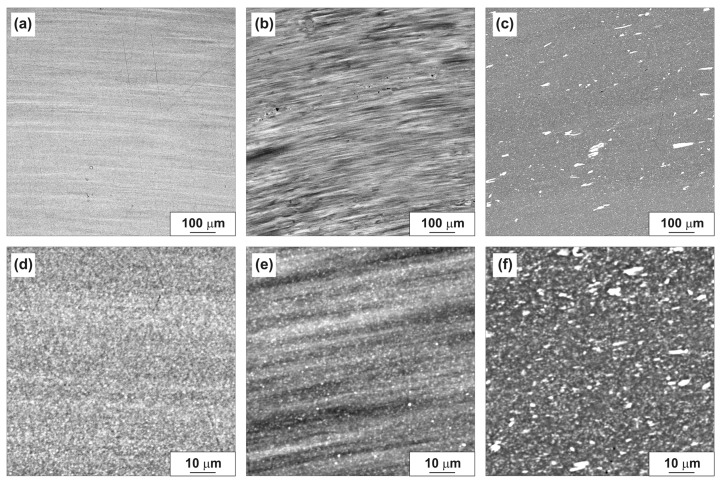
SEM images of the HPT-processed alloy surfaces: (**a**,**d**) Al–10% La; (**b**,**e**) Al–9% Ce; (**c**,**f**) Al–7% Ni.

SEM study of the HPT-processed specimen surface confirmed that as a result of HPT, the eutectic particles are crushed and more evenly distributed in the specimen volume (Figure 6). The most uniform distribution of particles is observed in the Al–10% La alloy. In the Al–6% Ni alloy, along with crushed eutectic particles, large fragments of primary eutectic particles are retained.

### 3.3. Effect of the HPT-Deformation on the Tensile Mechanical Properties of the Aluminum Alloys

**Figure 7:**
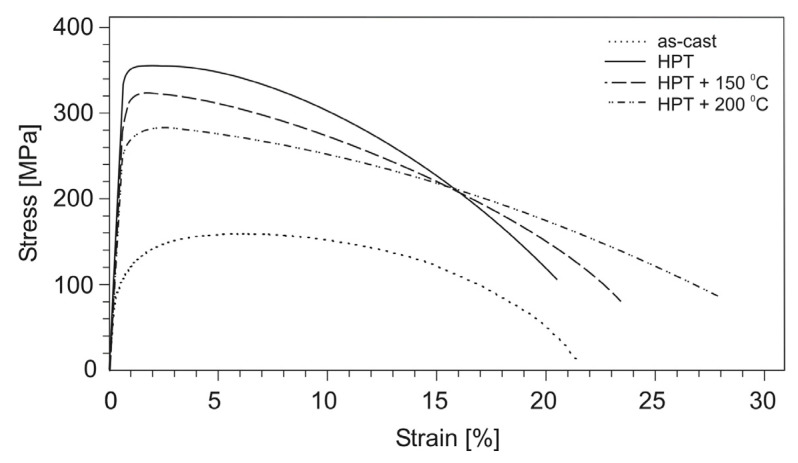
Stress–strain curves for tensile test specimens of the Al–10% La alloy in as-cast state, after HPT, and subsequent annealing.

**Figure 8:**
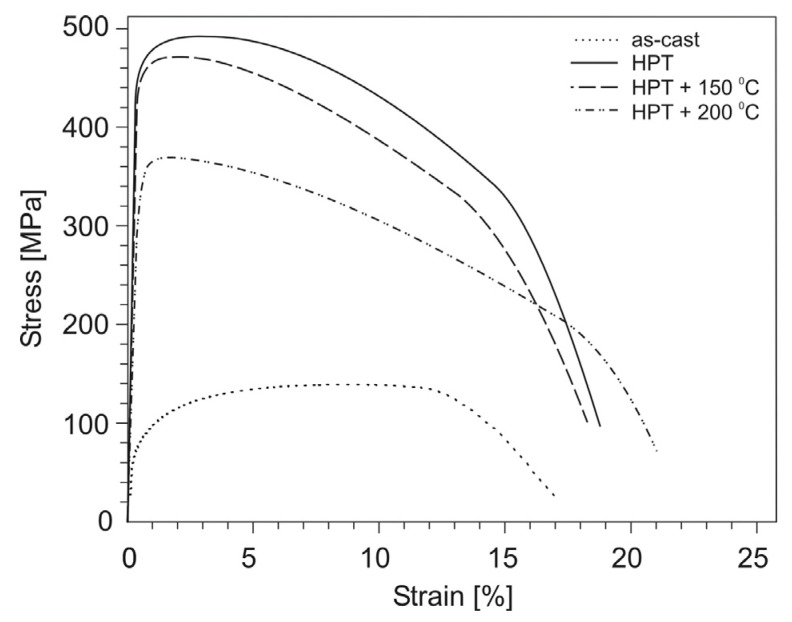
Stress–strain curves for tensile test specimens of the Al–9% Ce alloy in as-cast state, after HPT, and subsequent annealing.

**Figure 9:**
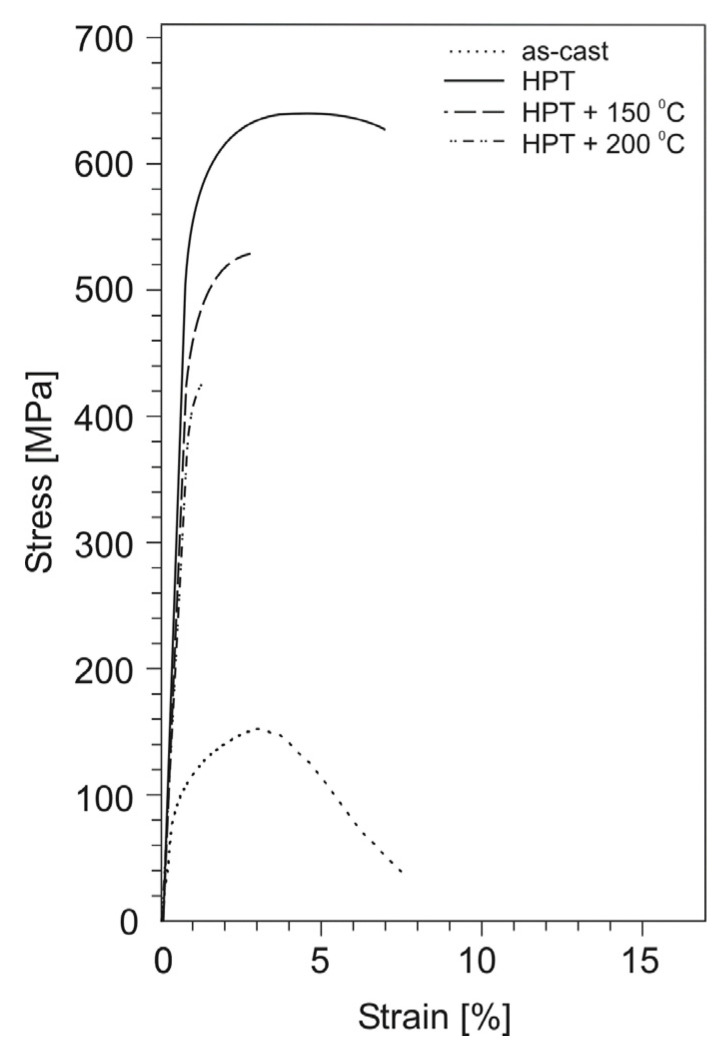
Stress–strain curves for tensile test specimens of the Al–7% Ni alloy in as-cast state, after HPT, and subsequent annealing.

The stress–strain curves for tensile specimens of aluminum alloys before and after HPT are shown in Figure 7, Figure 8 and Figure 9, and the values of mechanical properties are given in Table 2. As a result of HPT, a simultaneously multiple increase in the yield strength and ultimate tensile strength of all alloys with unchanged value (or slight decrease) of the relative elongation is observed. Thus, for the Al–10% La alloy, the yield and ultimate tensile strength increased from 113 to 347 MPa and from 173 to 358 MPa, respectively, i.e., 3 and 2 times, with an almost unchanged value of the relative elongation (22–20%). For the Al–9% Ce alloy, the yield strength and ultimate tensile strength increased from 75 to 456 MPa and from 135 to 495 MPa, respectively, i.e., 6 and 3.7 times, with an almost unchanged value of the relative elongation (17–18%). The highest absolute values of strength properties are observed for the Al–7% Ni alloy: the yield strength and ultimate tensile strength increased from 95 to 554 MPa and from 152 to 638 MPa, respectively, i.e., 5.8 and 4.2 times, with a decrease in the relative elongation from 8 to 5%.

As a result of HPT, the shape of the stress–strain curves for the Al–10% La and Al–9% Ce alloys changes, namely, the uniform strain stage significantly decreases and the localized strain stage increases. At the same time, the shape of the stress–strain curves for the Al–7% Ni alloy in as-cast state and after HPT differs less clearly.

The stress–strain curves of all cast alloys at the uniform deformation stage corresponded to the Hollomon’s Equation (2):

*S* = *K* × *e^n^* (2)

where: *S* and *e* are true stress and strain, respectively; *n*—strain hardening exponent.

So, for the Al–9% Ce alloy, the uniform strain stage is described by the equation *S* = 376.9 × *e*^0.42^ (determination coefficient *R*^2^ = 0.96), for the Al–10% La alloy—*S* = 485.8 × *e*^0.43^ (*R*^2^ = 0.96), and for the Al–7% Ni alloy—*S* = 935.5 × *e*^0.64^ (*R*^2^ = 0.99). However, the stress–strain curves of the HPT-processed alloys are more complex. The strain hardening exponent at the uniform deformation stage changes, namely, it decreases with an increase in the degree of strain. Simplistically, one can divide the uniform deformation stage into two stages and each approximate by the Hollomon’s equation. So, for the Al–9% Ce alloy, the initial section of uniform strain stage is described by the equation *S*_1_ = 2295 × *e*^0.55^ (*R*^2^ = 0.97), and the final one—*S*_2_ = 761.5 × *e*^0.14^ (*R*^2^ = 0.96). For the Al–10% La alloy, respectively, *S*_1_ = 1286.1 × *e*^0.42^ (*R*^2^ = 0.96) and *S*_2_ = 495.8 × *e*^0.09^ (*R*^2^ = 0.98). For the Al–7% Ni alloy, respectively, *S*_1_ = 4538.6 × *e*^0.79^ (*R*^2^ = 0.99) and *S*_2_ = 1329.9 × *e*^0.28^ (*R*^2^ = 0.95).

It is known that metallic nanostructured materials obtained by the SPD techniques are thermally unstable, especially metals with a low melting point, such as aluminum alloys [32]. Heating such materials to relatively low temperatures can lead to degradation of their mechanical properties. Therefore, the effect of annealing after HPT on the change in the mechanical properties of aluminum alloys was evaluated in this work. Annealing temperatures were chosen equal to 150 and 200 °C on the basis of literature data [10,20].

The stress–strain curves for tensile test specimens of the HPT-processed aluminum alloys after subsequent annealing are shown in Figure 7, Figure 8 and Figure 9, and the values of mechanical properties are given in Table 3.

Annealing had a different effect on the change in the mechanical properties of the HPT-processed aluminum alloys. So, for the Al–10% La alloy, annealing at a temperature of 150 °C led to a slight decrease in strength (by 8–11%), while the relative elongation increased from 20 to 24%. An increase in the annealing temperature to 200 °C led to a decrease in strength by 20–25% and an increase in elongation to 28%.

For the Al–9% Ce alloy, annealing at a temperature of 150 °C did not lead to a noticeable change in the mechanical properties (the strength decreased by less than 5%). An increase in the annealing temperature to 200 °C led to a decrease in strength by 25% and an increase in relative elongation to 22%.

On the contrary, annealing of the Al–7% Ni alloy already at a temperature of 150 °C led to a decrease in strength by 13–17% and a significant decrease in ductility (the relative elongation does not exceed 1%). An increase in the annealing temperature to 200 °C led to a decrease in strength by 22–32%, while the relative elongation decreased to 0%.

### 3.4. Fractographic Analysis of Aluminum Alloys after Tensile Testing

**Figure 10:**
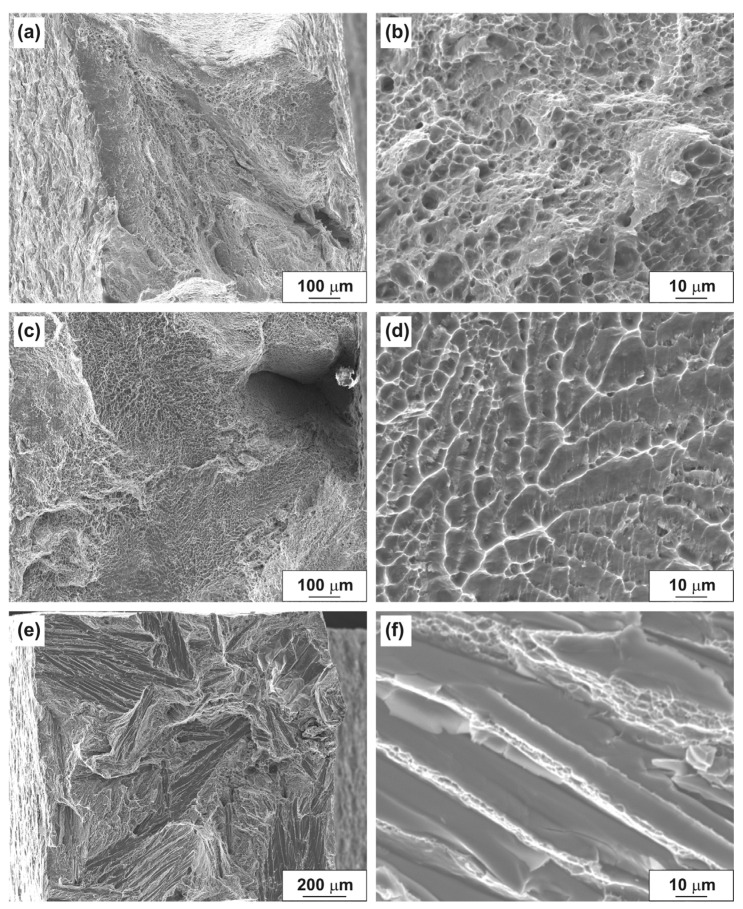
Fracture surfaces of aluminum alloys in as-cast state after tensile testing: (**a**,**b**) Al–10% La; (**c**,**d**) Al–9% Ce; (**e**,**f**) Al–7% Ni.

The fracture surfaces of aluminum alloys in as-cast state after tensile testing are shown in Figure 10. The fracture process of the tensile specimen of the Al–10% La alloy proceeds with the formation of significant reduction due to long-term localized strain (Figure 10a). The fracture is characterized predominantly by a ductile dimple fracture mechanism (Figure 10b). The fracture surface has a developed relief, which indicates a high energy intensity of fracture.

The fracture surface of the tensile specimen of the Al–9% Ce alloy is flat, which indicates a lower energy intensity of fracture in comparison with the Al–10% La alloy (Figure 10c). However, in this case, the predominantly ductile dimple microstructure of the surface fracture is also observed. In the fracture, a characteristic orientation of the dimples is observed, apparently corresponding to the initial dendritic structure of the ingot (Figure 10d).

The fracture surface of the tensile specimen of the Al–7% Ni alloy is the most flat, which indicates a low energy intensity of fracture in comparison with the Al–10% La and Al–9% Ce alloys (Figure 10e). The fracture mechanism is mixed; areas of ductile dimple fracture periodically alternate with areas of brittle fracture by the quasi-cleavage mechanism (Figure 10f). The formation of such an inhomogeneous fracture surface is apparently associated with the presence of large primary eutectic particles in the cast structure of the Al–7% Ni alloy.

**Figure 11:**
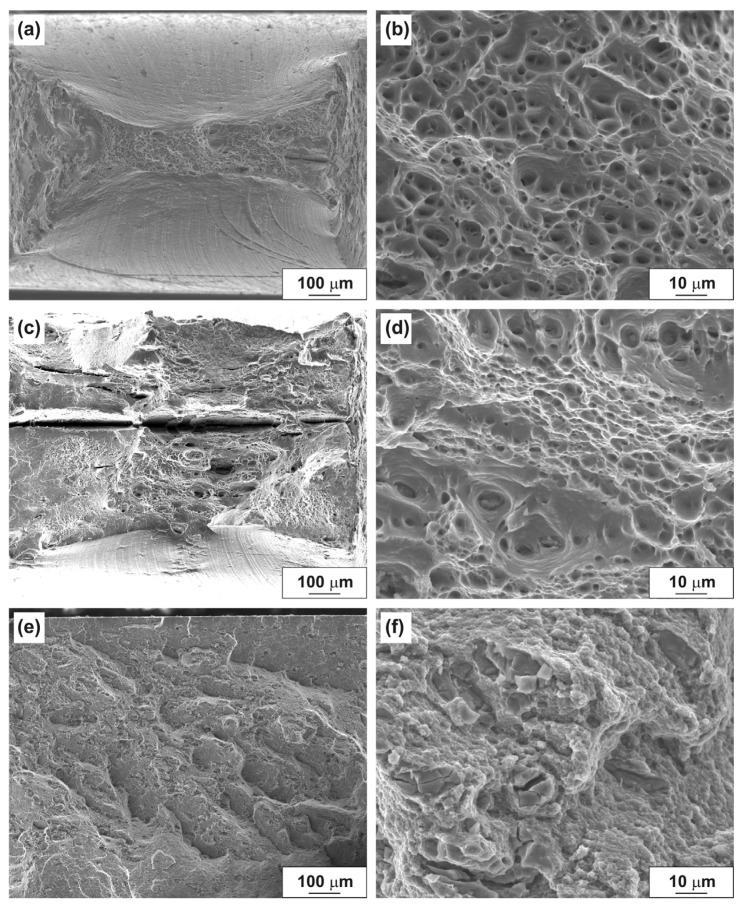
Fracture surfaces of the HPT-processed aluminum alloys after tensile tests: (**a**,**b**) Al–10% La; (**c**,**d**) Al–9% Ce; (**e**,**f**) Al–7% Ni.

The fracture surfaces of the HPT-processed aluminum alloys after tensile testing are shown in Figure 11. The reduction of the tensile specimen of the HPT-processed Al–10% La alloy is more significant than in as-cast state, which is the result of more prolonged localized strain (Figure 11a). The fracture of the tensile specimen, as well as in the as-cast state, proceeds mainly by the ductile dimple mechanism (Figure 11b).

The fracture surface of the tensile specimen of the HPT-processed Al–9% Ce alloy is flat, as well as in as-cast state (Figure 11c). The fracture mechanism is mixed; both areas of ductile dimple fracture and flat quasi-cleavage areas without a pronounced relief are observed (Figure 11d). A large number of secondary cracks with a length from 50 to 1500 μm (in the entire thickness of the tensile specimen) are also observed in the fracture.

The fracture surface of the tensile specimen of the HPT-processed Al–7% Ni alloy is the most flat, but the relief is more developed in comparison with the alloy in as-cast state (Figure 11e). The fracture mechanism is mixed: areas with numerous small (less than 1 μm) flat dimples and areas of brittle fracture by the quasi-cleavage mechanism are observed (Figure 11f).

It should be noted that in fractures of the HPT-processed Al–9% Ce and Al–7% Ni alloys, the oriented and periodic structures disappear. This is due to the formation of a more uniform structure of alloys during the HPT process, namely, the destruction of the inhomogeneous cast structure of the Al–9% Ce alloy and the crushing of large primary eutectic particles in the Al–7% Ni alloy.

## 4. Discussion

Some researchers adhere to the viewpoint that the process of structure formation under SPD and, in particular, under HPT, is cyclic [33]. An increase in the number of turns can lead to both the achievement of “saturation” in hardness, and vice versa, cause softening. In our study, the processing of all studied eutectic aluminum alloys was carried out with the number of turns *N* = 5. This number of turns was chosen to achieve a sufficiently uniform distribution of hardness (for the Al–10% La and Al–9% Ce alloys, a ‘dip’ in hardness was observed only in the central part of the specimens) and to compare the alloys with each other.

The observed in this study increase in hardness, as well as in the yield strength and ultimate tensile strength and maintaining of high plasticity of eutectic aluminum alloys, is associated both with the formation of nano- and submicrocrystalline structures in them and the eutectic particle refinement, and with the mechanism of structure formation during the HPT process.

As noted above, in TEM images of the structure of all HPT-processed aluminum alloys, there is a predominantly equiaxial shape of crystallites with the presence of triple intersections with an angle close to 120° and a specific contrast of bright-field images, which indicates a relatively low density of crystal defects in the structure of the alloys. The X-ray analysis data also confirm it (Table 1). The quantitative assessment of the increase in the yield stress of the samples due to the increase in the dislocation density (σ_d_) was carried out similarly to [25]; the results obtained are shown in Table 4.

At the same time, there are structure elements with both high-angle and low-angle boundaries. These signs may cautiously indicate that there is a mixed mechanism of structure formation, namely, dynamic recovery/recrystallization and fragmentation in alloys during the HPT process. The possibility of progress of the dynamic recrystallization process at low temperatures during SPD has been widely studied [32,33]. Thus, a structure with an ultrafine grain with a relatively low density of dislocations inside many crystallites was formed in the HPT-processed aluminum alloys. In this case, one can use the Hall-Petch relationship, according to which the increase in the yield stress is inversely proportional to the crystallite size: σ_0.2_ ~ 1/*d*^1/2^, where *d* is the crystallite size [34]. Thus, one of the factors for increasing the hardness and strength of alloys is the grain structure refinement to nano- and submicron sizes by the mechanism of dynamic recovery/recrystallization. The quantitative assessment of the increase in the yield stress in the samples using the Hall-Petch ratio (σ_H-P_) was carried out similarly to [25]; the results obtained are shown in Table 4. Note that the distribution of crystallite size in the alloy structures is not described by a normal distribution, therefore the average crystallite size is not an adequate factor characterizing the structure of the HPT-processed alloys (Figure 5). The use of an average crystallite size in calculations apparently leads to an inaccurate estimate of the theoretical hardening.

Simultaneously, a relatively low density of dislocations inside many crystallites increases the mean free path of dislocations, which in turn increases the total value of plastic deformation, i.e., provides a high value of the relative elongation. On the other hand, the plasticity of alloys can increase due to the refinement of large eutectic particle in cast structure, and by increasing the uniformity of their distribution in the sample volume (see TEM data in Figure 4).

The second factor in increasing the hardness and strength of alloys is the presence of nanosized particles of crushed eutectic with high hardness. The quantitative assessment of the increase in the yield stress of the samples due to disperse hardening by the Orowan mechanism (σ_Or_) was carried out similarly to [25]; the results obtained are shown in Table 4. At the same time, the accuracy of the calculation of strengthening by the Orowan mechanism seems to be low. This is due, firstly, to the fact that the structure of the alloys contains particles in a wide range of sizes, and secondly, the nature of the particles in the alloys is different (eutectic particles can be either cut or not cut by dislocations). Therefore, it is difficult to estimate the mean path length of dislocations between particles and other factors affecting the mechanism.

At the same time, we believe that the calculation and assessment of the contribution of individual strengthening factors (Orowan, dislocation, etc.) to the overall strengthening of alloys is often incorrect. In complex systems, which undoubtedly include severely deformed eutectic aluminum alloys, all strengthening mechanisms will interact with each other, which will not allow calculating their contribution to strengthening separately. In addition, the nature of eutectic particles has a serious impact on deformation process. The significant difference between the theoretical and experimental yield stress values for all alloys confirms the significantly more complex nature of strengthening in severely deformed eutectic aluminum alloys in comparison with the theoretical one.

Thus, the combination of all these structural transformations as a result of HPT (grain refinement, presence of defect-free grains, homogenization of the structure) contributes to a multiple increase in strength and to the preservation of high plasticity of the Al–10% La and Al–9% Ce alloys.

Given the lack of solubility of cerium, lanthanum and nickel in aluminum both in cast and HPT, all three alloys are two-phase, consisting of pure aluminum (alloy base) and eutectic: [(Al) + Al_11_La_3_]), [(Al) + Al_11_Ce_3_]), and [(Al) + Al_3_Ni], respectively, in the Al–10% La, Al–9% Ce, and Al–7% Ni alloys [4,6,35]. Since the volume fraction of the eutectic particles is close in all alloys—9–11%, the alloys differ mainly in the properties of the eutectic. The hardness of particles increases in the following order: Al_11_Ce_3_, Al_11_La_3_, and Al_3_Ni—3.5, 4.0, and 7.0–7.7 GPa, respectively [5,36,37,38]. In this case, the particle refinement process can occur simultaneously with the structure formation during SPD. Consequently, the presence of particles in the alloy structure has a major effect on the structure formation mechanism in alloys during HPT. It should be noted that the crushed eutectic particles in the structure of alloys subjected to HPT have a rounded shape, which indicates that their formation occurs with the participation of diffusion processes activated at high pressures.

It is interesting to note that despite the similar type of structure (qualitative and quantitative) of the HPT-processed Al–9% Ce and Al–10% La alloys, as well as the fact that the eutectic particles in the Al–9% Ce alloy have a slightly lower hardness than in the alloy Al–10% La, the HPT-processed Al–9% Ce alloy has a higher microhardness, higher strength, but less ductility under tension than the Al–10% La alloy. It can be assumed that the effect of the Al_11_La_3_ particles on structure formation during the HPT-deformation is different from the effect of the Al_11_Ce_3_ particles in the Al–9% Ce alloy. In this case, one should take into account, first, their different location and morphology in the cast structure, and secondly, the different orientation relationship between the lattice of the aluminum matrix and the lattice of the particle. It is known that with at a certain orientation ratio, one dislocation can slide in both phases simultaneously, i.e., the particle will be cut by the dislocation. The [$10\bar{1}$] Al || [001] Al11La3 orientation relationship between the phases in the Al–10% La alloy, apparently, is favorable for joint sliding. This facilitates the plastic deformation process in the Al–10% La alloy, which leads to its higher plasticity and lower strength than in the Al–9% Ce alloy.

In contrast, the Al–7% Ni alloy contains a large number of the hardest Al_3_Ni particles. Moreover, their predominant size after HPT is the smallest in comparison with the Al–10% La and Al–9% Ce alloys. Then these particles in the Al–7% Ni alloy will serve a dual role: on the one hand, they provide effective Orowan precipitation hardening, i.e., they provide high strength, and on the other hand, they reduce the plasticity by decrease in dislocations mean free path.

Thus, despite the fact that the crystallite size in the Al–7% Ni alloy is 2–3 times larger than in the Al–9% Ce and Al–10% La alloys, the HPT-processed Al–7% Ni alloy shows the greatest increase in strength and some decrease in relative elongation compared to cast alloy under tensile testing.

It can be noted that the level of strength achieved in the Al–10% La and Al–9% Ce alloys as a result of the HPT-deformation significantly exceeds the level of strength of the HPT-processed pure aluminum, low-alloy aluminum alloys and Al–Mg alloys [15,39], but inferior to the strength of the HPT-processed alloys alloyed with a solid solution, for example, Al–Cu–Mg–Mn system [40]. However, the strength level of the HPT-processed Al–Cu–Mg–Mn alloys is comparable to the strength level of the HPT-processed Al–7% Ni alloy. Thus, in [40], the strength of the HPT-processed Al–6Cu–0.7Mg–0.3Mn alloy reached 720 MPa. However, due to the fact that the high strength of such alloys is ensured by alloying the solid solution, the grain structure refinement to the nanoscale, and a significant increase in the dislocation density, the alloy had a very low plasticity.

It is also interesting to compare the results obtained with the scarce literature data on the HPT processing of similar eutectic aluminum alloys. Thus, in [25], a complex eutectic alloy Al–5.4% Ce–3.1% La (i.e., cerium was partially replaced by lanthanum) was processed by the HPT technique. As a result of HPT, a sixfold increase in strength was achieved with a twofold decrease in ductility. The achieved values of the yield strength and elongation were 475 MPa and 18%, respectively. The properties obtained are comparable to the Al–9% Ce alloy, but inferior (in terms of strength) to the Al–7% Ni alloy. It should be noted that the authors of [25] used a significantly larger number of turns (*N* = 20), which complicates a direct comparison of the results.

## 5. Conclusions

Based on the results of studying the effect of the HPT process (20-mm diameter specimens, *N* = 5, *P* = 6 GPa) on the microstructure and tensile mechanical properties of the Al–10% La, Al–9% Ce, and Al–7% Ni as-cast model binary eutectic aluminum alloys, the following conclusions were drawn:

(1) The HPT-deformation leads to the formation of nano- and submicrocrystalline structures in the Al–10% La and Al–9% Ce alloys and a submicrocrystalline structure in the Al–7% Ni alloy with a relatively low density of dislocations inside the crystallites, as well as to the eutectic particle refinement in all alloys. The predominant size ranges of crystallites in the Al–10% La, Al–9% Ce and Al–7% Ni alloys were 90–150 nm, 60–130 nm, and 170–400 nm, respectively, and eutectic particles were 10–50 nm, 10–40 nm, and 7–25 nm.
(2) A change in the structure of alloys as a result of HPT simultaneously leads to a multiple increase in their yield strength and ultimate tensile strength with unchanged value (or slight decrease) of the relative elongation. The highest absolute values of strength properties are observed for the Al–7% Ni alloy: the yield strength increased from 95 to 554 MPa (5.8 times), and the ultimate tensile strength from 152 to 638 MPa (4.2 times), respectively. The fracture of the HPT-processed alloys occurs by a ductile (Al–10% La alloy) or a mixed ductile-brittle mechanism (Al–9% Ce and Al–10% Ni alloys).
(3) The nature of eutectic particles has a significant effect both on the structure formation process in alloys during HPT and on the mechanical behavior of severely deformed alloys. Because of this, severely deformed alloys with a similar type of structure (Al–9% Ce and Al–10% La) have different levels of mechanical properties (strength and ductility).
(4) Annealing of the HPT-processed aluminum alloys in the temperature range 150–200 °C has a different effect on the change in their mechanical properties. The most noticeable change in the mechanical properties after annealing is observed for the Al–7% Ni alloy, the least noticeable for the Al–9% Ce alloy.

## Figures and Tables

**Table 1:** Results of X-ray studies of the eutectic aluminum alloys.

Alloy	Condition	2θ [°]	Lattice Parameter [Å]	FWHM [°]	Dislocation Density [m^−2^]
Al–10% La	as-cast	116.672	4.050	0.294	-
	HPT (*N* = 5)	116.745	4.049	0.491	1.3 × 10^14^
Al–9% Ce	as-cast	116.702	4.050	0.250	-
	HPT (*N* = 5)	116.744	4.049	0.525	1.9 × 10^14^
Al–7% Ni	as-cast	116.669	4.050	0.231	-
	HPT (*N* = 5)	116.724	4.049	0.504	0.6 × 10^14^

**Table 2:** Mechanical properties of eutectic aluminum alloys in as-cast state and after HPT.

Alloy	Microhardness, HV		Yield Strength, MPa		Ultimate Tensile Strength, MPa		Relative Elongation, %	
	as-Cast State	after HPT *	as-Cast State	after HPT	as-Cast State	after HPT	as-Cast State	after HPT
Al–10% La	52 ± 2	102 ± 3	113 ± 2	347 ± 3	173 ± 3	358 ± 3	22 ± 1	20 ± 1
Al–9% Ce	59 ± 5	142 ± 3	75 ± 2	456 ± 3	135 ± 3	495 ± 3	17 ± 1	18 ± 1
Al–7% Ni	65 ± 4	152 ± 3	95 ± 2	554 ± 4	152 ± 3	638 ± 4	8 ± 1	5 ± 1

* For the midradius of the specimen.

**Table 3:** Mechanical properties of the HPT-processed eutectic aluminum alloys after subsequent annealing.

Alloy	Annealing Temperature, °C	Yield Strength, MPa	Ultimate Tensile Strength, MPa	Relative Elongation, %
Al–10% La	150	308±5	326 ± 4	24 ± 1
	200	262 ± 5	285 ± 4	28 ± 1
Al–9% Ce	150	455 ± 6	480 ± 5	16 ± 1
	200	342 ± 5	371 ± 4	22 ± 1
Al–7% Ni	150	482 ± 5	527 ± 5	1 ± 0.5
	200	429 ± 5	433 ± 5	0

**Table 4:** Estimated contribution of different strengthening mechanisms to the increase in the yield stress of eutectic aluminum alloys after HPT.

Alloy	σ_0_ [MPa]	σ_H-P_ [MPa]	σ_Or_ [MPa]	σ_d_ [MPa]	σ_0.2_^theor^ [MPa]	σ_0.2_^exp^ [MPa]
Al–10% La	10	119	72	85	286	347
Al–9% Ce	10	138	74	103	325	456
Al–7% Ni	10	86	131	60	287	554

## Data Availability

The raw/processed data required to reproduce these findings cannot be shared at this time as the data also forms part of an ongoing study.
